# Sensation of pleasant touch: from molecules to circuits and behaviours

**DOI:** 10.1038/s41392-022-01161-1

**Published:** 2022-08-31

**Authors:** Ruining Hu, Rongfeng K. Hu

**Affiliations:** 1 Institute for Translational Brain Research, Fudan University, Shanghai, 200032 China; 2 Department of Psychology, Zhongshan Hospital, Institute for Translational Brain Research, State Key Laboratory of Medical Neurobiology, MOE Frontiers Center for Brain Science, Fudan University, Shanghai, 200032 China

**Keywords:** Neuroscience, Diseases

**Fig. 1:**
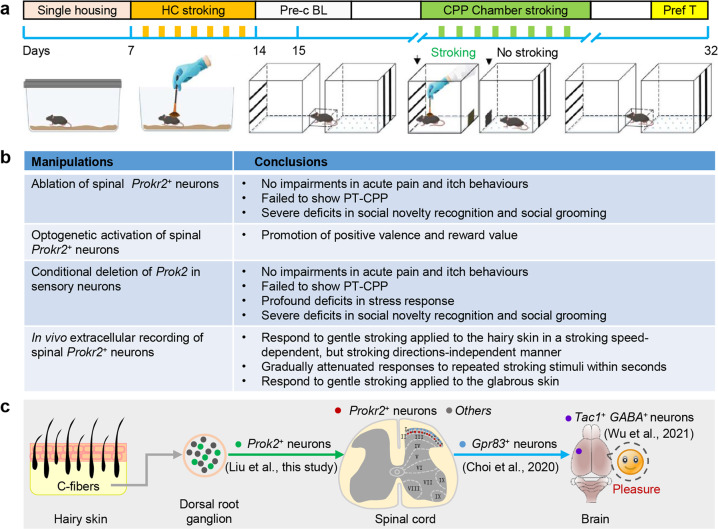
A brief summary of findings related to pleasant touch sensation. **a** Schematic diagram of the experimental procedure for PT-CPP. Modified from the highlighted paper.^1^ HC stroking, homecage stroking; Pre-c BL, preconditioning baseline; Pref T, preference test. **b** Main experiments and findings from the highlighted paper.^1^ **c** Diagrams of touch signalling from the skin to spinal cord and brain in recent studies^1–3^

A recent study by Liu et al*.* identified a crucial role of the PROK2-PROKR2 signalling within the spinal circuit in pleasant touch sensation (Fig. 1), providing the first evidence for the molecular and neural basis of how pleasant touch information is encoded and integrated from the skin to the spinal cord and highlighting the important role of neuropeptides in the processing of somatosensory information^1^ (Fig. 1).

Touch is essential for our interactions with the world around us. It is typically divided into two categories: discriminative touch and affective touch. Discriminative touch allows the one being touched to detect physical properties of tactile stimuli, such as location, texture and air pressure, whereas affective touch conveys emotional value regulated by social context. Pleasant social touch is a common form of prosocial comforting behaviour that plays important roles in the formation and maintenance of social bonds between animals, promoting affiliative, collaborative and sexual behaviour. However, the underlying neural mechanisms of pleasant touch are still largely unknown.

To address these questions, some attempts have been made. For example, a recent study by Wu et al. has identified a previously unknown role of the medial amygdala (MeA) in the encoding and control of allogrooming behaviour in mice. They further found a tackykinin-expressing subset of MeA GABAergic neurons that project to the medial preoptic area in the promotion of this behaviour.^2^ Another recent study by Choi et al. demonstrated that spinoparabrachial (SPB) neurons that express *Gpr83* convey tactile signals and that activation of these neurons can generate either positive or negative valence depending on stimulus intensity,^3^ revealing a previously unknown role of *Gpr83*^*+*^SPB neurons in affective touch.^3^ Together, these findings greatly deepen our understanding of neural mechanisms underlying pleasant touch (Fig. 1). However, the molecular and neural basis of how pleasant touch information is processed and signalled from somatosensory neurons to the spinal cord is still unexplored.

Because affective touch mediated by unmyelinated C fibers is a show process, the authors hypothesized that pleasant touch is encoded by slow-acting neuropeptides in C fibers and their associated excitatory G protein –coupled receptors (GPCRs) in the spinal cord, which together forms microcircuits to convey discrete sensory information from the skin to the brain.^4,5^ Interestingly, Liu et al. found that a previously uncharacterized subpopulation in the laminae II of the spinal cord that uniquely expresses prokineticin receptor 2 (PROKR2).^1^

To investigate the behavioural function of spinal *Prokr2*^+^ neurons, Liu et al. applied an intersectional genetic strategy to specifically ablate spinal *Prokr2*^+^ neurons in *Prokr2*-Cre mice, hereafter referred to as ABL mice.^1^ Surprisely, ABL mice did not display a statistically significant difference in response to diverse pain stimuli and itch-related behaviours, compared with the control mice.^1^ Numerous studies have implicated that the spinal cord play essential roles in the encoding and conveying of sensory information, including touch, pain and itch.^4,5^ Thus, the authors next sought to examine the role of spinal *Prokr2*^+^ neurons in touch sensation. As known, affective pleasant touch is considerably challenging to accurately infer and assess the affective state of mice. Liu et al. began to develop an unbiased two-chamber pleasant touch–conditioned place preference (PT-CPP) apparatus. They applied this apparatus to find that ABL mice could not develop the preference for stroking coupled chamber, suggesting that *Prokr2*^+^ neurons are required for transmitting pleasant touch.^1^ Moreover, optogenetic activation of *Prokr2*^+^ neurons could generate a strong place preference to the stimulation-coupled chamber in a real-time place preference test, demonstrating that *Prokr2*^+^ neurons encode reward.^1^

To explore neural correlates of pleasant touch, Liu et al. used an in vivo extracellular opto-tagging recording approach to investigate the neuronal dynamics of spinal *Prokr2*^+^ neurons in response to gentle stroking applied to the hairy skin of the hindlimb of ChR2-expressing *Prokr2*^cre^; Ai32 mice.^1^ They found that spinal *Prokr2*^+^ neurons respond most vigorously to gentle stroking in a manner that depends on stroking speed and that these neurons also exhibited comparable response strength, independent of stroking directions.^1^ The authors next examined whether spinal *Prokr2*^+^ neurons behave gradually attenuated responses to repeated stroking stimuli within seconds. The firing rate of spinal *Prokr2*^+^ neurons displayed fatigue to repeated brush stroking,^1^ indicating that the firing features of *Prokr2*^+^ neurons recapitulate the hallmarks of human CT fibers reinforces the notion that neural mechanisms of pleasant touch are conserved between humans and rodents. Together, these electrophysiological results further support the important role of spinal *Prokr2*^+^ neurons in the conveying and encoding of pleasant touch-related information.

Liu et al. next examined the role of PROK2 in pleasant touch by the generation of *Prok2* conditional knock-out mice (*Prok2* CKO mice). The authors first generated floxed *Prok2* mice and then bred them with *Na*_*v*_*1.8*^Cre^ mice to acquire *Prok2* CKO mice, which allows us to delete *Prok2* in *Na*_*v*_*1.8*^Cre^ neurons of dorsal root ganglions (DRGs). The results showed that mice with conditional knockout of *Prok2* in DRGs could not develop preference to stroking-coupled chamber in PT-CPP but nonetheless displayed normal pain and itch behaviours, highlighting the unique role of *Prok2* in pleasant touch rather than pain and itch.^1^

Pleasant social touch exhibits remarkable emotional benefits through indirectly activating brain reward-related circuits to release neuropeptides and neurotransmitters that encode prosocial value and anti-stress, thus serving to strengthen and maintain social bonding and helping to console distressed conspecifics. Thus, it is interesting and important to investigate whether a loss of pleasant touch sensation developmentally or in adult mice may lead to abnormal stress response. Liu et al. demonstrated that *Prok2* CKO but not ABL mice exhibited heightened stress response and anxiety-like behaviours,^1^ highlighting the importance of maternal touch in the development and health of offspring and further supporting the notion that early tactile experience plays an essential role in shaping the resilience of offspring in response to diverse stress.^5^

To sum, using a combination of approaches, including establishment of an unbiased behavioural paradigms, physiological tests, electrophysiology recording, optogenetics and genetics, and behavioural analysis, Liu et al. dissected the function of the PROK2-PROKR2 signalling within the spinal circuit in pleasant touch sensation.^1^ Notably, there are two major contributions of this research to the field. First, due to the lack of methodologies that allow us to accurate infer and assess of the affective state of animals experiencing pleasant touch, the development of the PT-CPP test in this study helps us overcome a major obstacle in the comprehensive understanding of neural basis of pleasant touch. Second, *Prok2* CKO mice generated in this research might serve as an invaluable animal model that can be used for studying the long-term effects of maternal touch on the development and health of offspring. Together, this study by Liu et al. not only provides us insights into understanding how pleasant touch information is encoded and transmitted from sensory neurons to the spinal cord across multiple levels, but also helps us develop alternative strategies for the innervations and treatment of neurological disorders associated with impaired social development and touch avoidance.

Still, many questions need to be answered. Does it exist other neuropeptides and/or molecules specifically for mediating pleasant touch? What are the spinal microcircuits that underlie pleasant touch sensation? How do neuropeptides synergically or independently mediate diverse somatosensory information (e.g., touch, pain and itch) in the spinal cord? Is it possible to develop more effective new systems for studying pleasant touch? Addressing these questions represents challenging but interesting future research directions in the field.
